# Differential Interaction between Invasive Thai Group B *Streptococcus* Sequence Type 283 and Caco-2 Cells

**DOI:** 10.3390/microorganisms10101917

**Published:** 2022-09-27

**Authors:** Siriphan Boonsilp, Marea Jikka Nealiga, Kinley Wangchuk, Anchalee Homkaew, Thanwa Wongsuk, Huttaya Thuncharoon, Paveesuda Suksomchit, Daranee Wasipraphai, Soraya Chaturongakul, Padungsri Dubbs

**Affiliations:** 1 Department of Clinical Pathology, Faculty of Medicine Vajira Hospital, Navamindradhiraj University, Bangkok 10300, Thailand; 2 Department of Microbiology, Faculty of Science, Mahidol University, Bangkok 10400, Thailand; 3 Microbiological Unit, Central Laboratory and Blood Bank, Faculty of Medicine Vajira Hospital, Navamindradhiraj University, Bangkok 10300, Thailand; 4 Laboratory Department, Taksin Hospital, Bangkok 10600, Thailand; 5 Molecular Medical Biosciences Cluster, Institute of Molecular Biosciences, Mahidol University, Nakhon Pathom 73170, Thailand; 6 Center for Emerging Bacterial Infections, Faculty of Science, Mahidol University, Bangkok 10400, Thailand

**Keywords:** group B *Streptococcus* (GBS), multilocus sequence typing (MLST), capsular polysaccharide (CPS) typing, Caco-2 cells, adhesion, invasion, intracellular survival, translocation assay

## Abstract

The emergence in Southeast Asia of invasive group B *Streptococcus* (GBS) infections in adults by sequence type (ST) 283 is suggested to be associated with fish consumption. Genotyping of 55 GBS clinical isolates revealed that 33/44 invasive isolates belonged to ST283/capsular polysaccharide type (CPS) III. This included 15/16 isolates recovered from younger adults aged 16–36 years. Seven ST283/CPSIII isolates from the blood, cerebrospinal fluid, or joint fluid were selected by the patient’s age at random to perform interaction studies with intestinal epithelial Caco-2 monolayers. The invasion efficiency profiles from this study classified these isolates into two groups; a higher invasion efficiency group 1 recovered from patients aged between 23 and 36 years, and a lower invasion efficiency group 2 recovered from the elderly and neonate. Intracellular survival tests revealed that only group 1 members could survive inside Caco-2 cells up to 32 h without replication. Additionally, all isolates tested were able to traverse across polarized Caco-2 monolayers. However, the timing of translocation varied among the isolates. These results indicated the potential of GBS invasion via the gastrointestinal tract and showed phenotypic variations in invasiveness, intracellular survival, and translocation efficiency between genetically closely related ST283 isolates infecting young adults and those infecting the elderly.

## 1. Introduction

Group B *Streptococcus* (GBS) or *Streptococcus agalactiae*, a Gram-positive coccus belonging to Lancefield’s serogroup B, often colonizes the lower gastrointestinal and genitourinary tracts of healthy adults [1]. In the 1970s, GBS was one of the leading causes of invasive infections among neonates [2]. During the past few decades, a shift in GBS invasive and noninvasive infections to nonpregnant adults has been reported, especially among the elderly and immunocompromised [3,4]. In Asia, a gradual increase in invasive GBS infections in immunocompetent adults has also been observed [5,6]. A prospective case-control study performed during a foodborne outbreak of GBS in Singapore revealed that GBS sequence type (ST) 283/serotype III was associated with invasive disease among nonpregnant adults, and had a tendency to infect younger adults with no underlying conditions compared to non-ST283 [7]. This ST has also been reported to cause invasive disease in humans and fish across Southeast Asia including Thailand [8], and its zoonotic potential has been proposed [9,10]. The transmission from fish to humans remains unproven, despite a few reports suggesting a phylogenetic link between GBS isolates from fish and humans [9,11]. The requirements for foodborne transmission include the ability of GBS to colonize intestinal epithelial cells, invade, and translocate across the epithelial barrier to other sites of the body to establish infections [12]. The human colorectal adenocarcinoma Caco-2 cell line (Caco-2 cells) has been used as a framework for invasion assays of numerous intestinal bacteria, including GBS, because of its ability to grow into a specialized monolayer of epithelial cells that resembles a sturdy barrier of human enterocytes [13,14]. A previous study showed that different GBS strains such as COH1 (CC17, serotype III) and 2603V/R (CC19, serotype V) differed in their ability to associate and invade differentiated Caco-2 cells [15]. Most studies of GBS cell interaction were mainly focused on invasive strains of varying genotypes that were recovered from neonates. The aim of this study was to perform comparative cell-interaction studies of selected isolates recovered mostly from adult patients using the Caco-2 cell line to gain insights into differential invasion behaviors among these isolates and to determine whether there is an association between the genotypes of GBS and the age of the infected patients.

## 2. Materials and Methods

### 2.1. Bacterial Isolates and Growth

A collection of 268 GBS isolates, recovered from patients who attended hospitals in Bangkok or nearby provinces during 2012–2016, consisted of 221 and 47 isolates from sterile and nonsterile body sites, respectively. Most isolates from sterile body sites were from blood (196), 7 from cerebrospinal fluid (CSF), 18 from joint fluid, while isolates from nonsterile body sites consisted of 3 from genital areas, 27 from pus samples, and 17 from urine. In cases where there were more than 1 isolate per patient, a single isolate was chosen to use in the study. Therefore, each of the 54 isolates used in this study originated from different patients and were selected according to the patient’s age and type of specimen. This includes 43 and 11 isolates from sterile and nonsterile body sites, respectively. One additional isolate, PK, was recovered from the blood of a young, previously healthy adult patient who had septic arthritis in 2018. Among these, 12 isolates; A5, A26, B105, B117, B140, C62, D23, D44, E4, E5, E19, and PK were published [11]. All isolates were verified by CAMP test and were tested for the presence of the *atr* gene [16]. Table S1 shows that, of the 55 isolates, 25 were recovered from blood, 12 from joint fluids, 7 from CSF, 5 from pus samples, 3 from swabs taken from genital areas and 3 isolates from urine samples. Patients were classified by age group. A total of 17 isolates were from the “elderly”, aged ≥60-years, 14 isolates were from “middle aged adults”, aged 40–59 years, 18 isolates were from “young adults”, aged 20–39 years, 2 isolates were from “adolescents”, aged 10–19 years, and 4 isolates were from “neonates”, aged 0–89 days.

Eight GBS/CPSIII invasive isolates recovered from blood, CSF, or joint fluid were chosen by the patient’s age at random to perform in vitro Caco-2 cell interaction studies Three isolates each were from young adults (B117, E19, and PK) and the elderly (B105, E5, and D23). The sole isolate of ST283/CPSIII recovered from a neonate (C22) was also included in this study plus the only ST1/CPSIII (A50) isolate. *Listeria monocytogenes* (*L. monocytogenes*) wild-type FSL-X1-0001 (LMWT) and *L. monocytogenes actA* deletion mutant (LM Δ*actA*) were used as the positive and negative controls, respectively [17].

All bacteria were grown on either blood agar plates or brain heart infusion agar/broth (BHI, Difco, Becton and Dickinson, Sparks, MD, USA) at 37 °C. The stock cultures were kept at −20 °C in 10% Difco skim milk (Becton and Dickinson, Sparks, MD, USA) containing 10% glycerol.

### 2.2. Genomic DNA Extraction

The bacterial pellet obtained from 5 mL of an overnight GBS culture was resuspended in 400 µL of TNE (10 mM Tris, pH 8.0, 10 mM NaCl, 10 mM EDTA) buffer, prior to the addition of 50 µL of lysozyme (80 mg/mL). The mixture was then incubated at 37 °C for 2 h before the addition of 75 µL of 10% sodium dodecyl sulfate. The bacterial lysate was incubated at 65 °C for 3 h. After phenol-chloroform extraction, 10 µL of RNase (20 mg/mL) was added to the lysate and incubated at 37 °C for 30 min After phenol-chloroform extraction, genomic DNA was ethanol precipitated. The DNA pellet was resuspended in 10 mM Tris buffer, pH 8, and stored at 4 °C. The concentration and purity of the genomic DNA were determined by Nanodrop (Denovix DS-11 FX+, Wilmington, DE, USA).

### 2.3. Multilocus Sequence Typing (MLST)

The DNA sequences of 7 housekeeping genes encoding alcohol dehydrogenase (*adhP*), phenylalanyl tRNA synthetase (*pheS*), glutamine transporter protein (*atr*), glutamine synthetase (*glnA*), serine dehydratase (*sdhA*), glucose kinase (*glcK*), and transketolase (*tkt*) were amplified according to the protocols described by Jones et al. [18], to obtain amplified products of approximately 672, 723, 627, 589, 646, 607, and 859 base pairs (bp), respectively. The purified amplified products obtained using GF-1 PCR Clean-up kit (Vivantis, Selangor Darul Ehsan, Malasia) were sent for DNA sequencing at Macrogen Inc. (Seoul, Korea) to sequence the internal region of each amplified product. The DNA sequences of the plus and minus strands were aligned using the SeqTrace program to verify the accuracy of the sequences, prior to analysis using the online BLASTn and BLASTx programs from the National Center for Biotechnology Information. After DNA sequence verification, the internal DNA sequences of the 7 housekeeping genes of each isolate were submitted to the MLST analysis website (http://pubmlst.org/sagalactiae/, accessed on 28 July 2022). The sequence type (ST) of each isolate was then assigned depending on its allelic profile. MLST clonal complexes (CCs) were predicted using the global optimal eBURST (goeBURST) algorithm. CCs were defined as groups of STs that differ from the common founder of the group by one or two alleles. A global population snapshot of GBS isolates was created to locate the STs generated in this study with the existing 1955 STs in the global MLST database using goeBURST implemented in the PHYLOViz2.0 software.

### 2.4. Phylogenetic Analysis

Phylogenetic analysis was conducted based upon the concatenated sequences of the seven loci obtained from the MLST. Mega10 software was used to create a dendrogram using an unweighted pair group method with arithmetic mean (UPGMA) with 1000 bootstrap replications.

### 2.5. Capsular Polysaccharide (CPS) Typing

CPS typing was performed using a multiplex PCR reaction as previously described [19]. Amplification reactions were performed using 50 ng of GBS genomic DNA with either primer mix I, consisting of specific primer pairs for identification of CPS types Ia, Ib, II, III, and IV or primer mix II containing specific primer pairs for identification of CPS types V, VI, VII, and VIII. The expected amplified products were 521 and 1826 bp for CPS Ia, 770 bp for CPS Ib, 397 bp for CPS II, 1826 bp for CPS III, 578 bp for CPS IV, 701 bp for CPS V, 487 bp for CPS VI, 371 bp for CPS VII, and 282 bp for CPS VIII.

### 2.6. Caco-2 Cell Culture

The Caco-2 cell line (ATCC HTB-37, ATCC, USA, passage number 13–25) was cultured in high glucose Dulbecco’s modified eagle medium (DMEM, Gibco, Life Technologies Corporation, Grand Island, NY, USA) without antibiotics, supplemented with 20% heat-inactivated fetal bovine serum (Gibco, Life Technologies Corporation, Grand Island, NY, USA), and 1% nonessential amino acids (Gibco, Life Technologies Corporation, Grand Island, NY, USA); and incubated with 5% CO_2_ at 37 °C. Cells used for the association, invasion, and cell survival assays were seeded at 2 × 10^4^ cells per cm^2^ in 24-well plates (Corning, Kennebunk, ME, USA), and grown for 15 days to allow differentiation. For translocation assays, the cells were seeded at 1 × 10^4^ cells per cm^2^ and grown on uncoated 3 µm pore size, 6.5 mm diameter, polycarbonate membrane inserts in a 24-well Transwell plate (Costar 3415, Corning, Kennebunk, ME, USA) for at least 21 days to obtain polarized cells. The media was changed every other day when cells were grown for infection experiments.

### 2.7. Quantitative Bacterial Interaction Assays

For in vitro infections, overnight grown cultures were used to inoculate fresh cultures that were grown to an OD_600_ of 0.2 to obtain approximately 5 × 10^7^ colony forming units (CFU)/mL. Bacterial suspensions were centrifuged and resuspended in culture medium to the desired multiplicity of infection (MOI). For each experiment, a colony count of the inoculum was performed to determine the actual number of infected bacteria (Table S2).

Bacterial association and invasion assays were performed as previously described [20] with slight modifications. Briefly, differentiated cell monolayers were infected with 1 mL of the bacterial inoculum at MOI 10. For association assays, infected cells were incubated for two hours, washed three times with 1× PBS, and then lysed with pH 11 water [21,22]. GBS survival in pH 11 water for 30 min was tested and the number of bacteria recovered from bacteria grown in 0.2% Triton-X-100 in 1× PBS and pH 11 water were comparable (Table S3). The number of associated bacteria, including both adhered and invaded, was determined by agar plating. Percent association was calculated by dividing the average number of associated bacteria by the average number of bacteria in the initial inoculum multiplied by 100. For invasion assays, the 2 h infected cells were washed three times with 1× PBS, before the extracellular bacteria were killed by further incubation for 2 h in media containing antibiotics (100 U penicillin and 100 µg/mL gentamicin). The infected cells at 4 h post infection (hpi) were then washed three times with 1× PBS before lysis with 1 mL cold, sterile pH 11 water to determine the number of invaded bacteria. Percent invasion was calculated by dividing the average number of intracellular bacteria by the average number of bacteria in the initial inoculum multiplied by 100. To ensure that all extracellular bacteria were killed after antibiotic treatment, the culture medium was collected, centrifuged at 9677× *g* for 5 min, and discarded. The pellet was then mixed with the remaining 100 µL of the supernatant before plating on an agar plate.

Bacteria surviving inside Caco-2 cells were quantified using a method similar to that of invasion assays. Caco-2 cell monolayers were infected with GBS for two hours, followed by two-hour antibiotic treatment. The 4-hpi infected cell monolayers were then washed three times with 1× PBS before the addition of antibiotic-free complete DMEM and further incubated up to 32-hpi. At the end of each time point (4-, 8-, 24-, and 32-hpi), the infected cells were washed three times with 1× PBS prior to cell lysis with 1 mL cold sterile pH 11 water, followed by agar plating. The number of intracellular bacteria was counted after an overnight incubation. Antibiotic-free media was used during this incubation period to avoid the possibility of antibiotic leakage into the cells and the killing of intracellular bacteria due to the prolonged exposure to antibiotics [23]. To confirm that there were no extracellular bacteria in the culture media at any of the time points, supernatant was collected, centrifuged and subsequently plated as in the invasion assays. The use of antibiotic-free media during bacterial survival assays yielded results that were similar to the results obtained using media containing antibiotics (Table S4).

To assess whether GBS was able to translocate from the apical to the basal compartment of Transwell plates, polarized Caco-2 cell monolayers, grown for at least 21 days, were infected at MOI 10. The inserts were transferred to new wells containing fresh media hourly to control bacterial multiplication at the bottom of the well. The apical media were also removed and replaced with fresh media every hour. Colony counting of bacteria was performed by immediately plating the media collected from the lower chamber at 2-, 4-, 6-, 8-, 10-, and 12-hpi.

### 2.8. Cell Viability Testing by Trypan Blue Exclusion Test

Cell viability in the control wells was assessed by trypan blue exclusion test for association and invasion experiments as well as intracellular survival test (Table S5). Briefly, Caco-2 cell monolayers were trypsinized by adding 0.1 mL of 0.25% trypsin-EDTA (Gibco, Life Technologies Corporation, Grand Island, NY, USA) followed by 5 min incubation in a 5% CO_2_ incubator at 37 °C. After incubation, the monolayers were gently rinsed with 0.2 mL of complete DMEM to remove the detached cells from the bottom of the wells. Aliquots of the trypsinized cells (0.l ml) were then added to 0.1 mL of 0.4% trypan blue (Gibco, Life Technologies Corporation, Grand Island, NY, USA). The numbers of both live and dead cells were counted using a hemocytometer as previously described [24]. Percent cell viability was determined by dividing the number of live cells (unstained by trypan blue) by the total number of cells (live and dead cells), and then multiplied by 100.

### 2.9. Transepithelial Electrical Resistance (TEER) Measurement

TEER was used as a quantitative method to measure cell barrier integrity. TEER values, in Ω.cm^2^, were measured using the Epithelial Volt/Ohm Meter, EVOM^2^ (World Precision Instruments, Sarasota, FL, USA) [25]. The percent change in TEER was calculated using the TEER measured at a given time point divided by the baseline TEER (the initial TEER value right after bacterial infection) minus 100 (%). TEER of uninfected Caco-2 cell monolayers was measured as a control.

### 2.10. Statistical Analysis

All assays were performed at least three times in triplicates (for association and invasion assays) or in duplicates (for translocation and intracellular survival assays). Statistical significance of data against the negative control was determined using student’s *t*-test at alpha 0.05 using the GraphPad Prism 7 program. Significant differences from the mean were determined using ordinary one-way ANOVA. The categorization of GBS isolates into groups using invasion efficiency profiles was determined by Tukey’s test (*p* < 0.05) using GraphPad Prism 7.

## 3. Results

### 3.1. Genotyping of GBS Isolates and the Relationship with Patients’ Age

**Figure 1:**
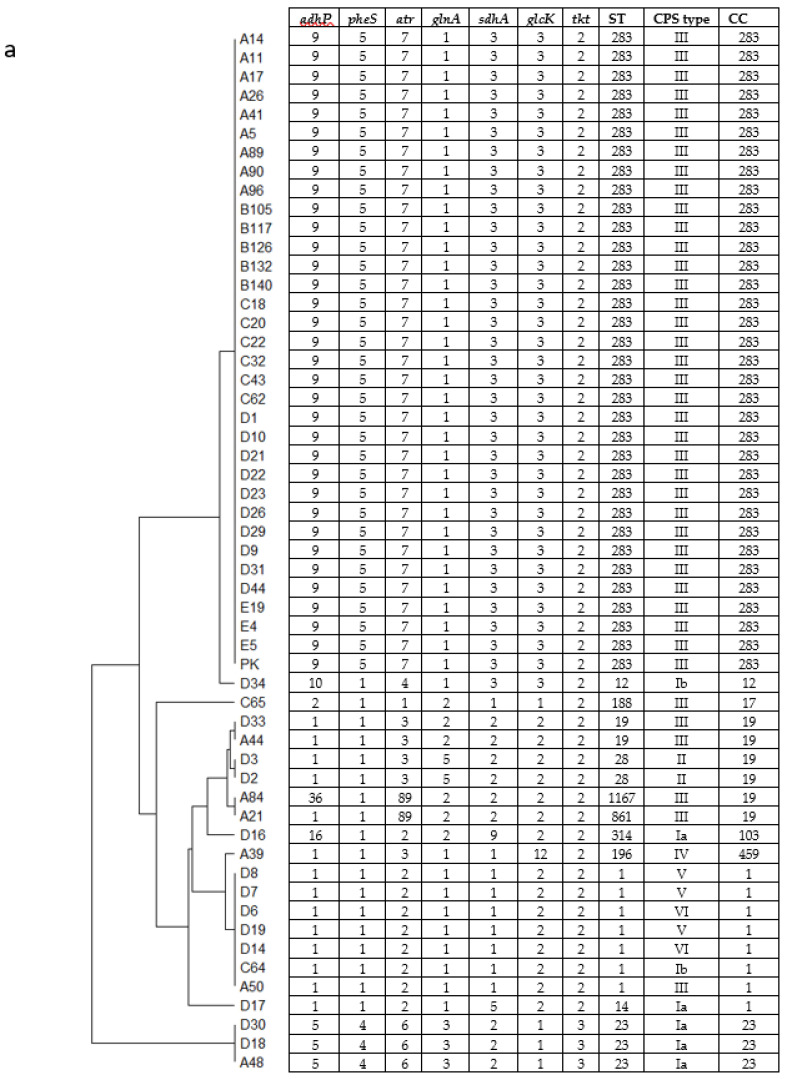

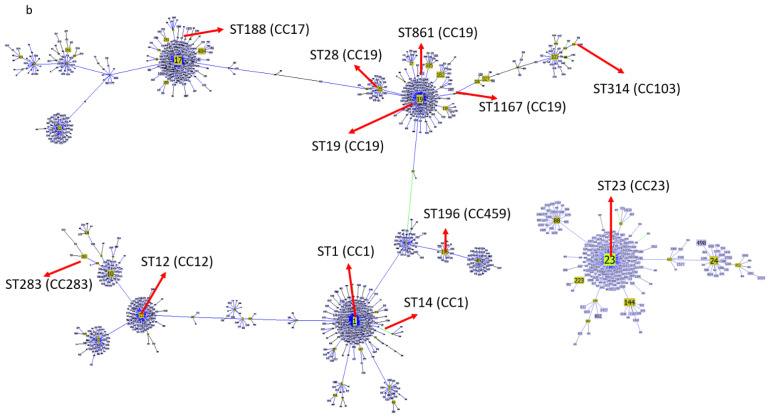
Dendrogram generated from 3456 base pairs concatenated multilocus sequence type (MLST) sequences of the 55 GBS isolates (**a**) and global optimal eBURST (goeBURST) analysis showing the clonal assignments of the obtained sequence types (**b**). MLST allelic profile, sequence type (ST), capsular polysaccharide (CPS) type, and clonal complex (CC) are also included in the dendrogram. As for goeBURST analysis, each ST is represented by a blue node. The central node represents the predicted ST founder shown in light green, while the subgroup founders are shown in yellow. The size of each node corresponds with the numbers of isolates in the STs database. Blue lines connect single-locus variants (SLV), and green lines connect double locus variants (DLV). CCs are identified by the number of the putative founder ST. Red arrows indicate the positions on the goeBURST analysis of each ST found in this study along with the corresponding CC in parenthesis.

MLST and CPS genotyping of 55 GBS clinical isolates from the central part of Thailand were performed. The MLST results classified our 55 isolates into 12 previously identified STs (Figure 1a). These 12 STs were assigned to 8 clonal complexes (CCs) including CC283 (34 isolates), CC1 (8 isolates), CC19 (6 isolates), CC23 (3 isolates), and 1 isolate each of CC12, CC17, CC103, and CC459 (Figure 1a). A population snapshot was created using our 12 STs along with the 1995 existing STs in the global MLST database using goeBURST (Figure 1b) to determine the link between our STs and the entire population available on the database. Both the prediction from the goeBURST (Figure 1b) and the phylogenetic dendrogram (Figure 1a) showed that our isolates are clustered into four major groups consisting of CC283, CC1, CC19, and CC23. The first 3 CCs (CC283, CC1 and CC19) are more closely linked evolutionarily, while CC23 is considered as an out group (Figure 1b). The present study showed that CC283 (ST283) is the predominant ST consisting of 34 out of 55 isolates (61.8%), followed by CC1 containing 8 isolates (14.5%) belonging to ST1, and ST14. CC19 consisted of 6 isolates (10.9%) belonging to ST19, ST28, ST861, and ST1167. The remaining CCs consisted of 1 isolate each of CC12 (ST12), CC17 (ST188, a single locus variant of ST17), CC103 (ST314, a double locus variant of ST103), and CC459 (ST196). As for CPS typing, a total of seven different CPS types were identified (Figure 1a). CPSIII was the most common (72.7%) amongst our isolates. CPSIa was the second most common CPS type (9.1%) found in this study consisting of CC23, CC1 (ST14), and CC103 (ST314). CPSIb consisted of CC1 and CC12. Both isolates belonging to ST28 (CC19) expressed CPSII. The single isolate belonging to ST196 (CC459), was identified as CPSIV, a common serotype for this ST [26]. The 3 isolates of ST1 (CC1) expressed CPSV and 2 expressed CPSVI. The results from the genotyping study not only showed that ST283/CPSIII was the predominant ST of the clinical GBS isolates tested, but also the main ST that caused invasive infections (33 out of 44 isolates, 75% from sterile body sites) (Table 1). Only 1 out of 11 isolates (9.1%) from nonsterile body sites belonged to this ST. In addition, GBS isolates belonging to ST283/CPSIII seemed to be able to infect patients from a variety of ages ranging from neonates to the elderly. Moreover, almost all GBS invasive isolates from young adults (14/15) including the only isolate from an adolescent, belonged to this particular ST (Table 1). Overall, 5 ST1 isolates expressing either CPSIb, III, V, or VI were recovered from sterile body sites and accounted for 11.6% of all invasive isolates (Table 1). Most of the neonate invasive isolates (3 out of 4) were caused by members of CC19 (ST28/CPSII, ST19/CPSIII, ST861/CPSIII) (Table 1). The remaining 1 invasive isolate from this CC (ST28/CPSII) was recovered from the joint fluid of a middle-aged adult. In this study, all 3 isolates belonging to CC23 (ST23) were recovered from non-sterile body sites only.

### 3.2. Comparison of Invasiveness among Selected CPSIII Isolates Using Caco-2 Cells

**Figure 2:**
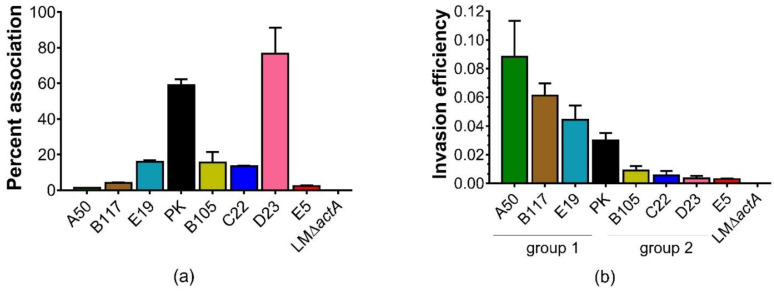

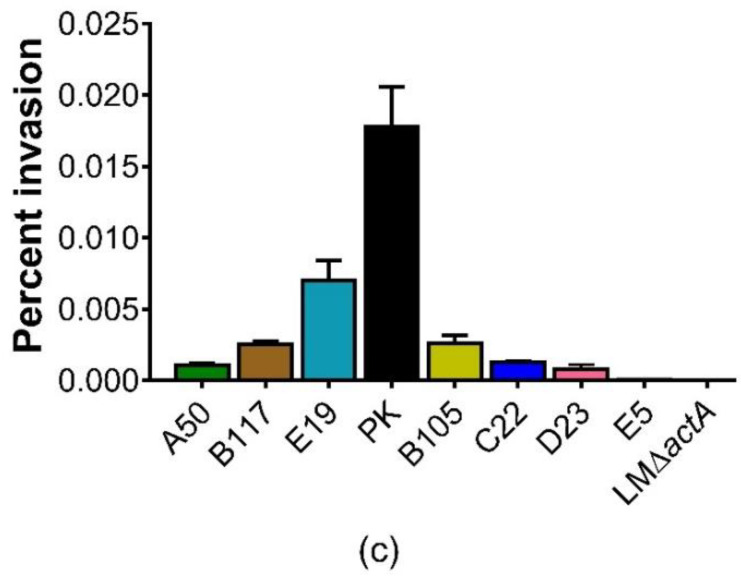
Percentage of cell association (**a**), invasion efficiency (**b**), and percentage of cell invasion (**c**) of various isolates of Group B *Streptococcus* (GBS). Differentiated Caco-2 cell monolayers were infected with GBS for 2 h at a multiplicity of invasion (MOI) of 10. The percent cell association is the number of associated bacteria (adhered and invaded cells) divided by the actual number of inoculated bacteria multiplied by 100. The invasion efficiency is calculated by the number of invaded bacteria (after 2 h of antibiotic treatment) divided by the number of associated bacteria (before antibiotic treatment) multiplied by 100. The percent cell invasion is the number of invaded bacteria divided by the actual number of inoculated bacteria multiplied by 100. Data on the *Y* axis are reported as the average number from three independent experiments with standard error bars. Assays were performed in triplicate. *Listeria monocytogenes* Δ*act*A (LM Δ*act*A) was used as a negative control.

The above results revealed that the predominant GBS ST283/CPSIII causes invasive infections in patients of various age groups, especially adults. We then compared the ability of ST283/CPSIII recovered from patients of different age groups to adhere and invade Caco-2 intestinal epithelial cells. A total of seven ST283/CPSIII invasive isolates (Table S1, isolates with asterisks) including three from young adults (B117, E19 and PK), three from the elderly (B105, D23, and E5), and one from a neonate (C22) were used. An additional CPSIII isolate from the second most dominant ST (ST1), A50, recovered from an elderly patient was also included in the study. The results showed that GBS attachment ability was variable among different isolates using one-way ANOVA (Figure 2a, *p* < 0.05). For example, PK (59%) and D23 (77%) had a significantly higher ability to adhere to Caco-2 cells than the rest of the isolates such as E19 (16%), B105 (16%), and C22 (13%), while a percent association well below 10% was observed for B117 (4%), E5 (2%), and 1% for A50 (Figure 2a).

Since the isolates have different capabilities to associate to Caco-2 cells, and in order to estimate the ability of these bacteria to invade differentiated Caco-2 cell monolayers, the invasion efficiency of individual isolates was calculated as a percentage ratio between the number of invaded bacteria and the number of associated bacteria [27,28]. Statistical analysis of the invasion efficiency profiles using Tukey’s test (*p* < 0.05) indicated that the CPSIII isolates could be categorized into two groups (Figure 2b). Group 1 consisted of A50, B117, E19, and PK with invasion efficiencies ranging from 0.03 to 0.088. Group 2 was comprised of B105, D23, E5, and C22 with invasion efficiencies that range from 0.003 to 0.009. Percent invasion was also determined by using a percentage ratio between the number of invaded bacteria and the number of bacteria in the initial inoculum. These results revealed that each isolate differed in its ability to adhere and invade the Caco-2 cells. Members of group 2, with a high percentage of cell attachment, such as D23, and to a lesser extent, B105 and C22, all had lower invasion efficiency (Figure 2b) and low percent invasion (Figure 2c). By contrast, members of group 1, such as A50 and B117, showed a low percentage of association (Figure 2a) and invasion (Figure 2b) had high invasion efficiency (Figure 2b). Interestingly, all group 1 members belonging to ST283 were obtained from younger patients aged 36 (B117), 28 (PK), and 23 years (E19). Meanwhile, the lower invasion efficiency members belonging to group 2 were ST283 isolates recovered from patients with compromised immune functions such as the newborn (C22) and the elderly patients (E5, D23 and B105). The isolate with the highest invasion efficiency was the ST1 isolate A50, recovered from an elderly patient.

### 3.3. Intracellular Survival of the GBS Isolates in Caco-2 Cells

**Figure 3:**
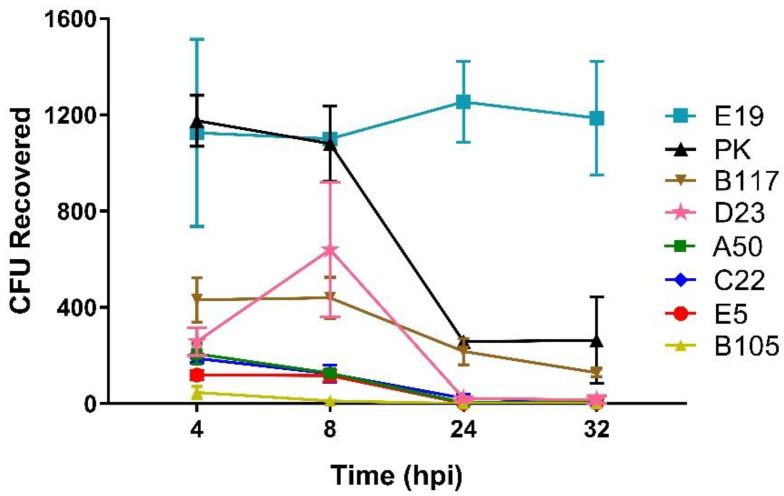
Time-course of intracellular survival of Group B *Streptococcus* (GBS) in Caco-2 cell monolayers. Differentiated Caco-2 cell monolayers were infected with GBS for 2 h at a multiplicity of infection of 10. After 2 h antibiotic treatment (4 h post infection, hpi), the infected cells were further incubated for an additional 8-, 24-, and 32-hpi. At each time point, the infected cells were lysed, and the number of intracellular bacteria were recovered. Isolates are labelled on the right. The data shown are the average number of intracellular bacteria in colony forming units (CFU) from three independent experiments with error bars representing standard error of measurement (± SEM). Assays were performed in duplicate.

The ability of Caco-2 epithelial cells to act as nonprofessional phagocytes by exerting oxidant-dependent bactericidal effects was reported [29,30]. Therefore, the survival of internalized GBS inside Caco-2 cells was assessed for up to 32-hpi. The experiments were performed as described in the invasion assays with further incubation of infected cells in antibiotic-free media for 8-, 24-, and 32-hpi prior to cell lysis. As expected, the results showed that for each isolate, the average number of intracellular bacteria at 4-hpi was consistent with those obtained from the cell invasion experiments. Moreover, E19 (light blue squares) could evade killing by Caco-2 cells until at least 32-hpi, in spite of its inability to multiply inside the cells. Intracellular survival of PK (black triangles) and, to a lesser extent, B117 (brown inverted triangles), was decreased at 24-hpi, but the average number of intracellular bacteria was sustained until 32-hpi (Figure 3). In contrast, all members of the less invasive group 2; D23 (pink stars), C22 (dark blue diamonds), E5 (red circles), and B105 (yellow triangles), including the one member of group 1, A50 (green squares), were almost entirely killed at 24-hpi. During this time-course experiment, >88% of the infected cells remained viable as assessed by a trypan blue exclusion test (Table S5).

### 3.4. Translocation of the GBS Isolates across Polarized Caco-2 Cell Monolayers

**Figure 4:**
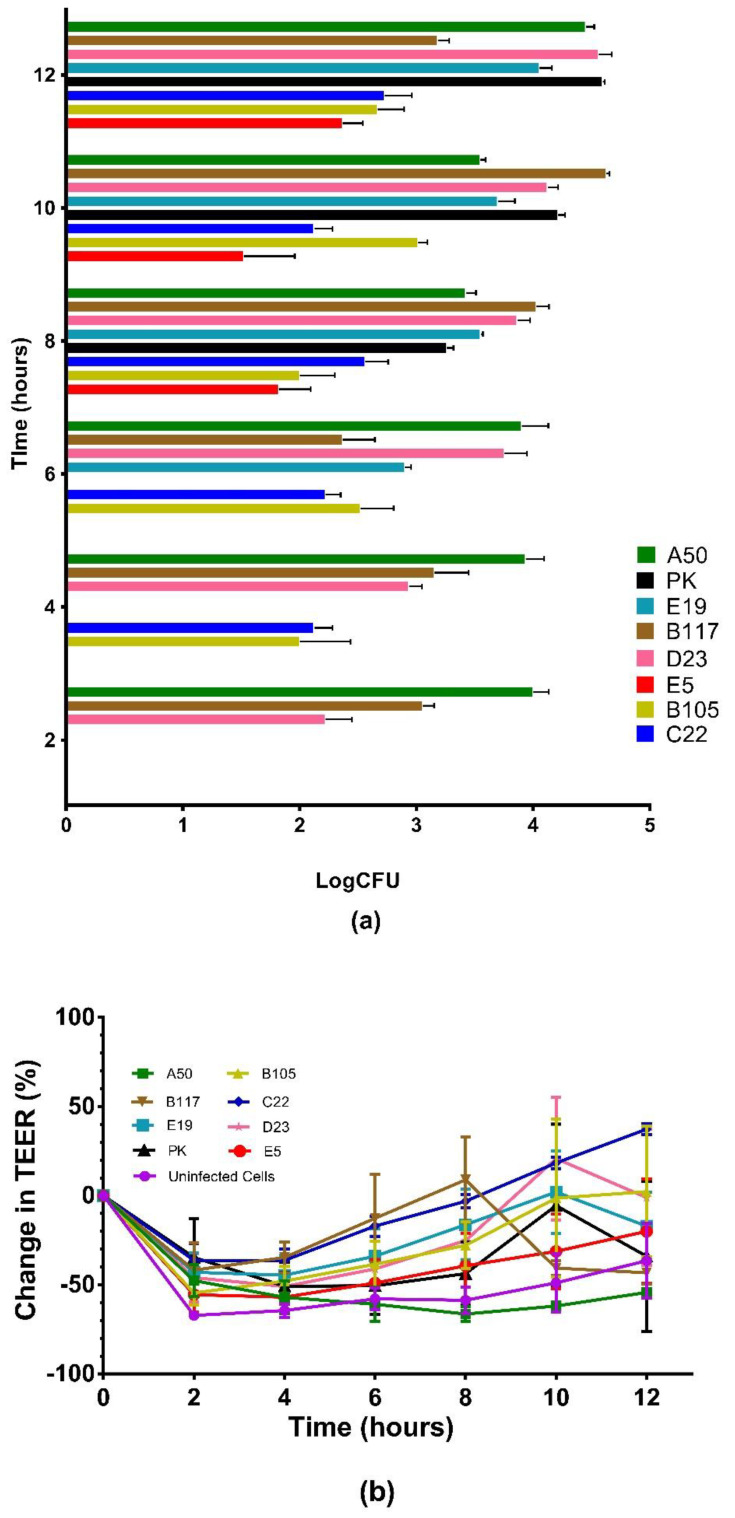
Time course of the number of translocated group B *Streptococcus* (GBS) across polarized Caco-2 cell monolayers after infection at a multiplicity of infection (MOI) of 10 (**a**) and percent changes in transepithelial electrical resistance (TEER) of corresponding infected cell monolayers at MOI 10 (**b**). Polarized Caco-2 cell monolayers grown in the Transwell plates infected with GBS group 1: A50 (green squares), B117 (brown inverted triangles), E19 (light blue squares), and PK (black triangles); group 2: B105 (yellow triangles), C22 (dark blue circles), D23 (pink stars), and E5 (red squares). The inserts were transferred to new wells containing fresh media every hour to reduce the effects of multiplication of the translocated bacteria in the bottom chambers. The translocated bacteria retrieved from the basal chambers at 2-, 4-, 6-, 8- and 12-h post infection (hpi) are shown in log colony forming units (LogCFU). Data shown are the mean averages from three independent experiments with error bars representing the standard error measurement (SEM). Assays were performed in duplicate. The TEER is measured at 2-h intervals. Percent TEER change is a ratio between the TEER of a certain time point and the initial TEER measured right after infection, minus 100. Each dot represents the means of percent TEER change with an error bar representing ±SEM. The percent TEER changes of uninfected cells are shown in purple circles.

In order to gage the ability of our GBS to translocate across the intestinal cell barrier, polarized Caco-2 cell monolayers grown on Transwell inserts were infected with GBS at MOI 10 and incubated for 12 h. During incubation, the inserts were transferred to new wells containing fresh media every hour to reduce the effect of the multiplication of the translocated bacteria in the bottom chambers. However, the bacteria were collected from the basal chambers for colony counting only at 2-, 4-, 6-, 8-, and 12-hpi. The results showed that all GBS isolates tested were able to translocate across polarized Caco-2 monolayers after 8-hpi. A50, B117, and D23 were able to translocate as early as 2-hpi, followed by C22 and B105 at 4-hpi (Figure 4a). We did not detect the translocated bacteria using the negative control, *Listeria monocytogenes* Δ*act*A (LM Δ*act*A). The number of translocated bacteria for all the isolates fluctuated over time. For example, the ranges of percent translocation (translocated bacteria divided by the initial inoculum multiplied by 100) of group 1 members were A50 (0.05–0.6), B117 (0.005–0.8), E19 (0.02–0.23), and PK (0.04–0.8). As for group 2 members, the ranges of translocation were B105 (0.002–0.02), C22 (0.003–0.01), D23 (0.003–0.7), and E5 (0.0007–0.005). The initial drop in percent change in TEER between 0 and 2 h for all bacterial isolates (Figure 4b) was most probably due to the disturbance of the Caco-2 cell monolayers from washing and adding bacterial samples after the initial measurements. A gradual increase in percent change in TEER over the succeeding hours was observed in all ST283/CPSIII isolates (Figure 4b). This may be due to the presence of bacteria on the apical side of the Transwell inserts which resulted in the regulation of Caco-2 cell morphology and tight junctions to limit the passage of bacteria across the epithelial barrier [31]. Alternatively, the fluctuation of TEER values observed among some strains may have been due to the addition of fresh media every hour prior to TEER measurements.

## 4. Discussion

GBS remains a main cause of serious neonatal invasive infections in several countries, despite the implementation of intrapartum prophylaxis [32,33]. The reported incidence of neonatal GBS sepsis in Thailand was low (about 0.12/1000 live births) [34], despite a 12–18% prevalence of maternal GBS colonization [35,36,37]. This low incidence of GBS infection in neonates was also reported from other Asian countries especially in Southeast Asia [38,39]. A 12-year retrospective study from 2004 to 2015 obtained from two hospitals in Bangkok, Thailand, revealed that invasive GBS infections were higher among the elderly and middle-aged adults compared to neonates (Figure S1). This reflects a potential health threat to older adults from GBS invasive disease [40]. There are five major CCs of human GBS circulating around the globe; CC1, CC10, CC17, CC19, and CC23 [18]. The hypervirulent, CC17/CPSIII, is a major CC responsible for neonatal invasive infections worldwide [18,33,41,42,43], while CC1/serotype IV and V as well as CC23/serotype IV have been associated with GBS invasive disease in nonpregnant adults in North America [44,45]. Genotyping results showed that a major cause of GBS invasive disease found in patients in the central region of Thailand was ST283/CPSIII (75%, 33 from a total of 44 invasive isolates from 40 adults and four neonates). These results were consistent with a previous report of 76% (102/139) of ST283 recovered from GBS bloodstream infections in patients from hospitals in the eastern region of Thailand [8]. Based on a high frequency of ST283 causing invasive disease of adult patients in several Thai hospitals, it is conceivable that ST283 might be responsible for the majority of GBS invasive infections in Thai adults.

The spread of ST283 in humans is more or less restricted to Southeast Asia [8]. In addition, an assessment of GBS invasive patients during an outbreak in Singapore in 2015 indicated that 35.8% (146 out of 408) were infected by GBS ST283. Most of these patients were younger with fewer pre-existing medical conditions, as compared to patients with non-ST283 invasive infections [46]. Likewise, in this study, almost all (15 out of 16) invasive isolates recovered from patients aged between 16 to 36 years old belonged to ST283. The tendency of this ST to cause invasive disease in patients of various ages, including younger adults, implies that this genotype might have adapted to overcome more potent immune responses of the host. Phylogenetic analysis using whole genome sequences of ST283 recovered from patients of different ages from Southeast Asian countries, including the 12 invasive isolates in this study (see materials and methods), revealed that the genome sequences of isolates from Thailand, Laos, and Singapore are clustered within the same clade [11], implying that these isolates are genetically closely related.

GBS invasive infections can cause clinical manifestations such as sepsis, septic arthritis, and meningitis [47]. An outbreak in Singapore linked the cause of ST283 infection to raw freshwater fish consumption [10]. Moreover, the consumption of fish has been reported as one of the risk factors for GBS colonization [48]. A previous study showed that GBS colonizers in the gut could potentially invade the intestinal barriers and pass into the bloodstream of the patient [49]. GBS invasion could occur via multiple means such as actin-dependent endocytosis mediated either through a surface protein like alpha C protein [50] or caveolar lipid rafts [13].

Through our cell interaction studies, we showed that all our invasive isolates invade Caco-2 cell monolayers with distinct invasive behaviors. For example, a poor correlation was found between the adhesion capability and invasion efficiencies in some isolates, such as D23, A50, B117, B105, and C22, even though adhesion is a prerequisite for bacterial invasion (Figure 2a,b). With regard to the invasion profiles, we found disparities among isolates of ST283/CPSIII (Figure 2b). The higher invasive isolates (group 1) consisted of isolates recovered from young adults (E19, B117, and PK), while the lower invasive isolates were retrieved from the elderly (D23, B105, and E5) and a neonate (C22). We also discovered a decrease in intracellular viability over a 24-hpi period for isolates belonging to group 2 (D23, C22, E5, and B105) as well as in one group 1 isolate belonging to ST1 (A50) (Figure 3). This suggested that isolates recovered from patients with lower immune function, such as the elderly and neonate, were unable to resist oxidant-dependent intracellular killing by Caco-2 cells [29]. However, since the number of isolates tested in this study was small, future experiments with larger numbers of isolates are needed in order to prove this notion. Conversely, we found that all ST283 isolates belonging to group 1: E19 and, to a lesser extent, PK and B117, could resist Caco-2 intracellular killing for up to 32-hpi, but were unable to replicate inside the cells (Figure 3). Likewise, GBS ST17 strain COH1 could survive in A549 monolayers for up to 8-hpi without replication [51]. This capability of intracellular survival might protect the organism from host defense responses and antibiotics [51,52], and may serve as a reservoir for subsequent spreading, as has been suggested for the intracellular survival of Group A *Streptococcus* within cultured epithelial cells [53].

Bacterial pathogens can translocate across polarized epithelial cells via either transcellular or paracellular routes [54]. It was demonstrated by transmission electron microscopy that GBS strain COH1 at MOI 100 could traverse Caco-2 cells via a paracellular route to the basolateral membrane without dislocation of ZO-1 (the tight junction scaffolding protein) [15]. Using translocation assays at MOI 10, we have shown that all invasive isolates tested had the ability to translocate across an epithelial cell layer by 8-hpi (Figure 4b). The ability for translocation was not associated with bacterial cell invasion since both members of group 1 and group 2 were able to efficiently translocate polarized Caco-2 monolayers. This could be due to a higher expression of host receptors for GBS in polarized Caco-2 cells growing on the Transwells. A previous study showed that the timing for apical expression of CFA receptors specific for enterotoxigenic *Escherichia coli* binding was dependent on the differentiation process of Caco-2 cells [55]. Since all tested isolates are invasive, this implied that GBS isolates may disseminate into the bloodstream by traversing across polarized cell monolayers quickly to avoid intracellular killing by host cells. Unlike the COH1 strain, we have no evidence whether these isolates traverse Caco-2 cells via the transcellular and/or paracellular route(s).

## 5. Conclusions

Taken together, the results of this study highlight the potential of intestinal GBS to cause invasive infection in humans. Although whole genome sequences of isolates in our study, such as E19, PK, B117, B105, and E5, were clustered with other Thai ST283 isolates [11], cell interaction studies using Caco-2 cell monolayers showed that these isolates were phenotypically different in their magnitudes of invasiveness, their capacity for intracellular survival, and their capability to translocate across polarized Caco-2 cell monolayers. These discrepancies between ST283 isolates could be in part due to the differential expression of genes involved in pathogenicity [12]. Moreover, certain isolates, especially E19, may possess a yet unknown mechanism that allows these isolates to survive intracellularly within Caco-2 cells.

## Figures and Tables

**Table 1:** Distribution of 55 selected Group B *Streptococcus* (GBS) classified by isolate source, age of disease onset, sequence types (ST), and capsular polysaccharide (CPS) types.

Patients’ Age	Total No. of Patients	No. of Patients (ST/CPS of GBS)					
		Sterile Body Site			Non-Sterile Body Site		
		CSF	Blood	Joint Fluid	Pus	Urine	Genital Area (Swab)
Neonates (0–89 d)	4	1 (ST283/III) 1 (ST861/III)	1 (ST28/II) 1 (ST19/III)	0	0	0	0
Adolescents (10–19 y)	2	0	1 (ST283/III)	0	0	0	1 (ST1167/III)
Young adult (20–39 y)	18	2 (ST283/III)	9 (ST283/III) 1 (ST314/Ia)	3 (ST283/III)	1 (ST1/V)	1 (ST23/Ia)	1 (ST196/IV)
Middle aged adults (40–59 y)	14	1 (ST283/III)	4 (ST283/III) 2 (ST1/V)	2 (ST283/III) 1 (ST188/III) 1 (ST28/II) 1 (ST1/Ib)	1 (ST1/VI)	1 (ST19/III)	0
Elderly (≥60 y)	17	2 (ST283/III)	4 (ST283/III) 1 (ST1/III) 1 (ST1/VI)	4 (ST283/III)	2 (ST23/Ia) 1 (ST14/Ia)	1 (ST12/Ib)	1 (ST283/III)

GBS: Group B *Streptococcus*; CSF: Cerebrospinal Fluid.

## Data Availability

Not applicable.

## Supplementary Materials

The following supporting information can be downloaded at: https://www.mdpi.com/article/10.3390/microorganisms10101917/s1, Table S1: Patients’ data, type of specimens and collection date of 55 group B *Streptococcus* (GBS) isolates used in the study; Table S2: Average number of actual bacterial inoculum used for adhesion and invasion (a) as well as translocation (b) assays; Table S3: Comparison of group B *Streptococcus* (GBS) survival in pH 11 water versus 0.2% Triton-X 100 in 1× PBS (a) as well as comparison between number of GBS intracellular survival after Caco-2 cell lysis using pH11 water (I) and 0.2% Triton-X in 1×PBS (II) in bacterial intracellular survival assays (b); Table S4: Comparison of Group B *Streptococcus* (GBS) intracellular survival (a) and percent Caco-2 cell survival (b) during bacterial intracellular survival assays when incubated with antibiotic-free media versus media containing antibiotics; Table S5: Cell viability as assessed by trypan blue exclusion test for bacterial association and invasion assays (a) as well as bacterial intracellular survival assays (b); and Figure S1: Retrospective data showing age distribution of invasive Group B *Streptococcus* isolated from patients attending two hospitals in Bangkok, Thailand during 2004–2015.
