# Supplementary material for: Differential Interaction between Invasive Thai Group B Streptococcus Sequence Type 283 and Caco-2 Cells
Source: Microorganisms. 2022 Sep 27;10(10):1917. doi: 10.3390/microorganisms10101917 (PMC9611625; doi:10.3390/microorganisms10101917)
Supplement: Supplementary file 1 [file microorganisms-10-01917-s001.zip › Figure S1.pdf]

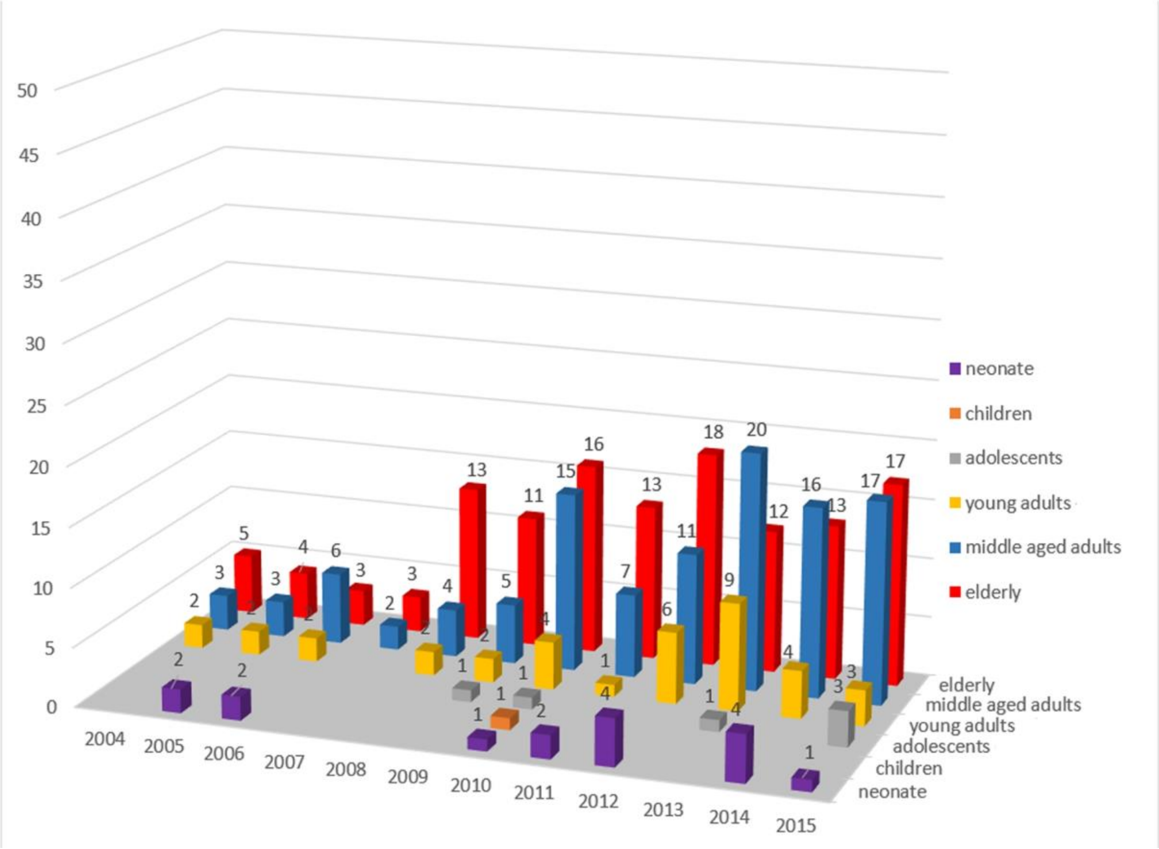

Figure S1. Retrospective data showing age distribution of invasive Group B *Streptococcus* isolated from patients attending two hospitals in Bangkok, Thailand during 2004–2015.
