# Supplementary material for: Differential Interaction between Invasive Thai Group B Streptococcus Sequence Type 283 and Caco-2 Cells
Source: Microorganisms. 2022 Sep 27;10(10):1917. doi: 10.3390/microorganisms10101917 (PMC9611625; doi:10.3390/microorganisms10101917)
Supplement: Supplementary file 1 [file microorganisms-10-01917-s001.zip › Table S1.pdf]

Table S1: Patients' data, type of specimens and collection date of 55 group B *Streptococcus* (GBS) isolates used in this study

No.	GBS isolate	Collection date (month/year)	Type of Specimen	Patient's Age (years)/Sex (male or female)	Age group classification
1	A5	10/12	Blood	16/F	Adolescence
2	A11	11/12	Joint fluid	82/M	Elderly
3	A14	10/12	Joint fluid	64/F	Elderly
4	A17	11/12	Blood	25/M	Young adult
5	A21	10/12	CSF	2 months/M	Neonate
6	A26	10/12	Blood	32/F	Young adult
7	A39	12/12	Vaginal swab	30/F	Young adult
8	A41	11/12	Joint Fluid	37/M	Young adult
9	A44	02/12	Blood	2 months/M	Neonate
10	A48	10/12	Wound swab	80/M	Elderly
11	A50*	05/12	Blood	91/F	Elderly
12	A84	05/13	Cervical swab	15/F	Adolescence
13	A89	05/13	Blood	22/M	Young adult
14	A90	05/13	Blood	31/M	Young adult
15	A96	05/13	Joint fluid	38/F	Young adult
16	B105*	03/13	Joint fluid	69/M	Elderly
17	B117*	05/13	Blood	36/M	Young adult
18	B132	05/13	Blood	31/M	Young adult
19	B140	06/13	Blood	48/F	Middle aged adult
20	C18	08/13	Joint (wrist)	31	Young adult
21	C20	08/13	Joint (wrist)	46/F	Middle aged adult
22	C22*	08/13	CSF	0 day/F	Neonate
23	C32	09/13	Joint fluid	58/F	Middle aged adult
24	C43	09/13	Joint fluid (knee)	75/F	Elderly
25	C62	12/13	CSF	24/M	Young adult
26	C64	11/13	Joint fluid	58/M	Middle aged adult
27	C65	12/13	Joint Fluid (knee)	54/M	Middle aged adult
28	D1	02/14	Blood	76/F	Elderly
29	D2	04/14	Joint fluid (knee)	51/M	Middle aged adult
30	D3	01/14	Blood	22 days/M	Neonate
31	D5	04/14	Joint fluid	30/M	Young adult
32	D6	04/14	Blood	63/F	Elderly
33	D7	04/14	Blood	40/M	Middle aged adult
34	D8	05/14	Blood	45/M	Middle aged adult
35	D9	05/14	Blood	44/F	Middle aged adult
36	D10	05/14	Blood	67/F	Elderly
37	D14	03/14	Pus (Foot)	53/F	Middle aged adult
38	D16	04/14	Blood	39/M	Young adult
39	D17	03/14	Pus (Foot)	72/F	Elderly
40	D18	04/14	Urine	36/F	Young adult
41	D19	03/14	Pus	39/M	Young adult
42	D21	05/14	Blood	59/M	Middle aged adult
43	D22	05/14	Blood	25/M	Young adult
44	D23*	05/14	Blood	86/F	Elderly
45	D26	05/14	Blood	58/F	Middle aged adult
46	D29	05/14	Blood	74/M	Elderly
47	D30	05/14	Pus	71/M	Elderly
48	D31	05/14	Scrotum swab	63/M	Elderly
49	D33	05/14	Urine	59/F	Female
50	D34	05/14	Urine	65/F	Elderly
51	D44	07/14	CSF	47/M	Middle aged adult
52	E4	05/16	CSF	60/M	Elderly
53	E5*	05/16	CSF	69/F	Elderly
54	E19*	09/16	CSF	23/F	Young adult
55	PK*	08/18	Blood	28/M	Young adult

M = Male, F = Female, CSF = Cerebrospinal fluid

* = Isolates used in cell interaction studies
