# Supplementary material for: Differential Interaction between Invasive Thai Group B Streptococcus Sequence Type 283 and Caco-2 Cells
Source: Microorganisms. 2022 Sep 27;10(10):1917. doi: 10.3390/microorganisms10101917 (PMC9611625; doi:10.3390/microorganisms10101917)
Supplement: Supplementary file 1 [file microorganisms-10-01917-s001.zip › Table S2.pdf]

Table S2: (a) Average number of actual bacterial inoculum used for adhesion and invasion assays

GBS Isolate	Average CFU/ml
A50	1.2×10^7
B105	1.1×10^7
B117	1.0×10^7
C22	1.7×10^7
D23	9.5×10^6
E5	1.4×10^7
E19	1.6×10^7
PK	1.5×10^7
L. monocytogenes Δ*act*A	1.5×10^7

(b) Average number of actual bacterial inoculum used for translocation Assays

GBS Isolate	Average CFU/ml
A50	5.6×10^6
B105	4.5×10^6
B117	5.5×10^6
C22	4.7×10^6
D23	5.6×10^6
E5	4.6×10^6
E19	4.7×10^6
PK	4.3×10^6
L. monocytogenes Δ*act*A	5.0×10^6
