# Supplementary material for: Differential Interaction between Invasive Thai Group B Streptococcus Sequence Type 283 and Caco-2 Cells
Source: Microorganisms. 2022 Sep 27;10(10):1917. doi: 10.3390/microorganisms10101917 (PMC9611625; doi:10.3390/microorganisms10101917)
Supplement: Supplementary file 1 [file microorganisms-10-01917-s001.zip › Table S3.pdf]

Table S3: (a) Comparison of group B *Streptococcus* (GBS) survival in pH 11 water versus 0.2% Triton-X 100 in 1× PBS

Methodology:

1. GBS strain D23 was grown to Log phase (~3 hours) in BHI broth in 37ºC with shaking

2. 1mL of bacterial suspension was placed in microtubes and centrifuged at 4000 rpm in 4 minutes

3. bacterial pellets were re-suspended with either pH11 water or 0.2% Triton-X in 1× PBS

4. Suspensions (triplicates for both conditions) were placed in wells of tissue culture plates (12-well) and incubated at 37ºC

5. After 30 minutes of incubation, the bacterial suspensions were diluted and plated for colony counting

	Replicate no.	Independent Assay		
**pH11 water**		**A**	**B**	**C**
	**1**	$1.5×10^7$	$1.6×10^7$	$1.7×10^7$
	**2**	$1.8×10^7$	$1.9×10^7$	$1.8×10^7$
	**3**	$1.4×10^7$	$1.2×10^7$	$1.9×10^7$
	**Average**	$1.6×10^7$	$1.6×10^7$	$1.8×10^7$

	Replicate no.	Independent Assay		
**0.2% Triton-X in PBS**		**A**	**B**	**C**
	**1**	$2.3×10^7$	$1.3×10^7$	$1.9×10^7$
	**2**	$2.0×10^7$	$1.6×10^7$	$2.8×10^7$
	**3**	$2.3×10^7$	$2.5×10^7$	$2.3×10^7$
	**Average**	$2.2×10^7$	$1.8×10^7$	$2.3×10^7$

- Averages between bacterial survival in pH 11 water and 0.2% Triton X in 1× PBS are not significant according to student's t-test (P>0.05).

(b) Comparison between number of GBS intracellular survival after Caco-2 cell lysis using pH11 water (I) and 0.2% Triton-X in 1× PBS (II) in bacterial intracellular survival assays

Intracellular Survival (recovered GBS in CFU) after various hours post infection (hpi)

GBS isolate		4-hpi	8-hpi	24-hpi	32-hpi
**B117**	**I**	80	85	65	12
	**II**	35	40	80	25

- Mean averages between I and II are not significant according to student's t-test (P>0.05).
- The intracellular survival assays were performed as described in the materials and methods.
