# Supplementary material for: Differential Interaction between Invasive Thai Group B Streptococcus Sequence Type 283 and Caco-2 Cells
Source: Microorganisms. 2022 Sep 27;10(10):1917. doi: 10.3390/microorganisms10101917 (PMC9611625; doi:10.3390/microorganisms10101917)
Supplement: Supplementary file 1 [file microorganisms-10-01917-s001.zip › Table S4.pdf]

Table S4: Comparison of Group B *Streptococcus* (GBS) intracellular survival (a) and percent Caco-2 cell survival (b) during bacterial intracellular survival assays when incubated with antibiotic-free media versus media containing antibiotics

GBS isolates B117 and PK at MOI 0.1 were infected to Caco-2 cell monolayers and incubated for up to 32 hours post infection (hpi) using similar methods as bacterial intracellular survival assays. The following results show the number of intracellular bacteria (a) as well as the percent survival of Caco-2 cells (b) in media containing antibiotics (**I**) and in antibiotic-free media (**II**) per given time point.

(a)

**Average Intracellular Bacterial Survival**					
**GBS isolates**		**4-hpi**	**8-hpi**	**24-hpi**	**32-hpi**
**B117**	**I**	10	9	8	12.5
	**II**	8	8.5	6.5	12
**PK**	**I**	2.5	1.5	7.5	4
	**II**	3.5	2	9.5	5

(b)

**Percent Cell Survival (%)**					
**GBS isolates**		**4-hpi**	**8-hpi**	**24-hpi**	**32-hpi**
**B117**	**I**	97.32	94.73	95.57	86.67
	**II**	95.04	97.43	93.57	91.49
**PK**	**I**	94.82	93.65	91.67	92.45
	**II**	95.65	91.11	93.42	87.72
**Control**	**I**	94.11	92.98	93.10	87.72
	**II**	90.47	95.31	95.18	87.93

Control – uninfected Caco-2 cells

I – Caco-2 cells incubated with antibiotic media

II – Caco-2 cells incubated with antibiotic-free media

- Percent GBS survival and percent cell survival obtained from both conditions per time point were not statistically significant as determined by student's t-test ($P>0.05$).
