# Supplementary material for: Differential Interaction between Invasive Thai Group B Streptococcus Sequence Type 283 and Caco-2 Cells
Source: Microorganisms. 2022 Sep 27;10(10):1917. doi: 10.3390/microorganisms10101917 (PMC9611625; doi:10.3390/microorganisms10101917)
Supplement: Supplementary file 1 [file microorganisms-10-01917-s001.zip › TableS5.pdf]

Table S5: Cell viability as assessed by trypan blue exclusion test for bacterial association and invasion assays (a) as well as bacterial intracellular survival assays (b)

(a) Data for Caco-2 cell viability assays (via trypan blue exclusion) after infection and subsequent antibiotic treatment at MOI 10 with A, B, and C representing three independent assays

**A50**				
	**Live cells**	**Dead cells**	**Total number of cells**	**% Viability**
A	264	1	265	99.62%
B	232	4	236	98.31%
C	274	1	275	99.64%
**B105**				
	**Live cells**	**Dead cells**	**Total number of cells**	**% Viability**
A	216	5	221	97.74%
B	146	4	150	97.33%
C	158	3	161	98.44%
**B117**				
	**Live cells**	**Dead cells**	**Total number of cells**	**% Viability**
A	369	3	372	99.19%
B	352	5	357	98.60%
C	344	4	348	98.85%
**C22**				
	**Live cells**	**Dead cells**	**Total number of cells**	**% Viability**
A	195	3	198	98.48%
B	208	2	210	99.05%
C	246	2	248	99.19%
**E5**				
	**Live cells**	**Dead cells**	**Total number of cells**	**% Viability**
A	139	5	144	96.52%
B	218	10	228	95.61%
C	213	5	218	97.71%

E19				
	**Live cells**	**Dead cells**	**Total number of cells**	**% Viability**
A	95	7	102	93.14%
B	92	11	103	89.32%
C	98	8	106	92.45%

PK				
	**Live cells**	**Dead cells**	**Total number of cells**	**% Viability**
A	135	5	140	96.44%
B	119	4	123	96.75%
C	182	5	187	97.33%
**D23**				
	**Live cells**	**Dead cells**	**Total number of cells**	**% Viability**
A	129	6	135	95.56%
B	118	10	128	92.19%
C	107	5	112	95.53%
***L. monocytogenes* Δ*actA* mutant (negative control)**				
	**Live cells**	**Dead cells**	**Total number of cells**	**% Viability**
A	114	6	120	95.00%
B	161	7	168	95.83%
C	185	7	192	96.35%

(b) Data for Caco-2 cell viability assays (via trypan blue exclusion) for intracellular survival assays at MOI 10 up to 32 hours post-infection

4 hours	**Live cells**	**Dead cells**	**Total number of cells**	**% Viability**
A50	97	2	99	97.98%
B105	66	4	70	94.29%
B117	65	1	66	98.48%
C22	98	2	100	98.00%
D23	90	3	93	96.77%
E5	96	3	99	96.975
E19	81	2	83	97.59%
PK	72	1	73	98.63%
LM Δ*act*A	63	2	65	96.92%

8 hours	**Live cells**	**Dead cells**	**Total number of cells**	**% Viability**
A50	32	2	34	94.12%
B105	55	0	55	100.00%
B117	40	5	45	88.89%
C22	85	4	89	95.50%
D23	96	2	98	97.96%
E5	79	5	84	94.05%
E19	103	4	107	96.56%
PK	86	3	89	96.63%
LM Δ*act*A	36	4	40	90.00%

24 hours	**Live cells**	**Dead cells**	**Total number of cells**	**% Viability**
A50	29	4	33	87.88%
B105	26	3	29	89.66%
B117	37	6	42	88.10%
C22	114	12	126	90.48%
D23	56	3	59	94.92%
E5	88	6	94	93.62%
E19	82	5	87	94.25%
PK	61	4	65	93.85%
LM Δ*act*A	28	3	31	90.32%

32 hours	**Live cells**	**Dead cells**	**Total number of cells**	**% Viability**
A50	38	1	39	97.46%
B105	33	3	36	91.67%
B117	35	4	39	89.74%
C22	134	6	140	95.71%
D23	36	2	38	94.74%
E5	88	4	92	95.65%
E19	52	6	58	89.66%
PK	69	6	75	92.00%
LM Δ*act*A	26	2	28	92.86%
